# Selective estrogen receptor modulators and deep venous thrombosis after an emergent operation in senior women

**DOI:** 10.1186/s40981-023-00665-1

**Published:** 2023-11-03

**Authors:** Keiko Kishida, Masanobu Furukawa, Masayuki Nakashima, Idumi Kubota, Yukio Hayashi

**Affiliations:** 1 Phamaceutics, Yoka Municipal Hospital, Yabu, Hyogo 667-8555 Japan; 2 Clinical Llaborateory, Yoka Municipal Hospital, Yabu, Hyogo 667-8555 Japan; 3 Quality Mmanagament, Yoka Municipal Hospital, Yabu, Hyogo 667-8555 Japan; 4 Anesthesiology Service, Yoka Municipal Hospital, Yabu, 1878-1, Yoka, Yoka-cho, Hyogo 667-5555 Japan

**Keywords:** Drugs: Selective estrogen receptor modulators, Complication: Deep venous thrombosis, Patients: Aged female, Operation: Emergent

## Abstract

**Background:**

Selective estrogen receptor modulators (SERMs), clinically applied to osteoporosis, may have potential risk of deep venous thrombosis (DVT) and discontinuation of SERMs may be required before surgery. However, we cannot discontinue SERMs for a certain duration, when patients undergo an emergent operation.

**Case presentation:**

We reported two aged patients undergoing an emergent orthopedic surgery for lower extremities while taking SERMs for osteoporosis before the operation. DVT was newly developed in one patient and worsened in the other patient after the operation. We found eight aged patients underwent the same operation while taking SERMs for recent 3 years, including the two cases and DVT did not occur in the other six patients. Thus, the incidence of DVT in our patient population was 25%.

**Conclusion:**

We showed that DVT developed or worsened after operation in two patients taking SERMs before operation. Ultrasound examination after operation may be recommended in these population. (149 words).

## Introduction

Deep venous thrombosis (DVT) in the lower extremities is a relatively rare complication after surgery. However, it has the potential to develop into a pulmonary embolism. Thus, prophylaxis of DVT after surgery is an important issue during the anesthetic management of a surgical patient.

It is well known that an oral contraceptive pill is a risk factor of DVT. Thus, the Medical Eligibility Criteria for Contraceptive Use (MEC) 5th edition by WHO [1] recommended that oral contraceptive pills should be discontinued for a certain duration before major surgery. Selective estrogen receptor modulators (SERMs), which were clinically applied to osteoporosis, have a similar pharmacological properties with oral contraceptive pills [2]. Thus, this medicine has the potential risk of DVT, and discontinuation of SERMs may be required before surgery [3]. However, when aged patients who take SERMs for osteoporosis have to undergo an emergent operation, we cannot discontinue SERMs for a certain duration. We report two aged patients who underwent an emergent operation while taking SERMs for osteoporosis before the operation. DVT was newly developed after the operation in one patient and worsened after the operation in the other patient.

## Case reports

### Case 1

**Fig. 1:**
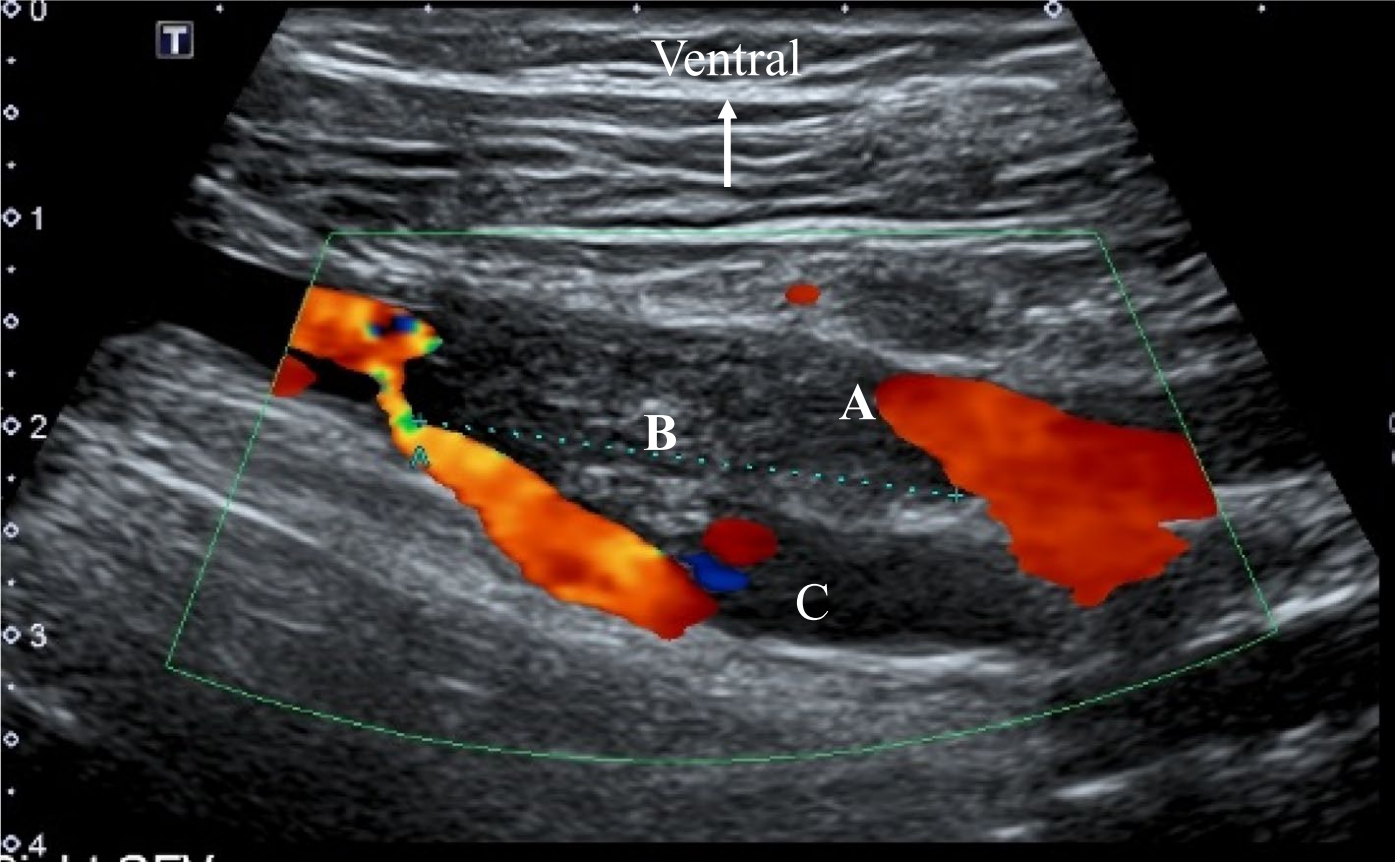
Ultrasound image of the right leg, longitudinal view in case 1. A color Doppler image shows an intraluminal filling defect with no flow in the right femoral vein (**A**) and the vein contains the intraluminal material (**B**) indicating deep venous thrombosis. **C** deep femoral vein. A small white arrow shows the direction of this image

An 85-year-old female (146 cm, 54 kg) was admitted to our hospital because of a periprosthetic infection. She underwent right total knee replacement 6 years ago and the replacement because of a periprosthic infection 3 years ago. Her past history included hypertension and diabetes, which were controlled with medications. Also, she took bazedoxifene for osteoporosis. Debridement was emergently scheduled under general anesthesia. In our hospital, an ultrasound examination of the lower extremities was performed routinely in patients undergoing orthopedic surgery for the lower extremity before the operation and on the seventh postoperative day. Her preoperative ultrasound examination of her lower extremities showed DVT in the left soleal vein. Anesthesia was induced with propofol followed by rocuronium to facilitate tracheal intubation and maintained with sevoflurane and remifentanil in oxygen/air. Monitoring in the operating room included ECG, noninvasive blood pressure, percutaneous oxygen saturation, end-tidal carbon dioxide concentration, and rectal temperature. The operation was uneventful, the trachea was extubated and the patient was returned to the ward. The duration of surgery was 1 h and 15 min and the duration of anesthesia was 1 h and 59 min. The ultrasound examination for lower extremities on the seventh postoperative day showed DVT in the bilateral soleal and the right femoral veins (Fig. 1). We started anticoagulation therapy with warfarin immediately, resulting in early lysis of DVT with no serious consequences such as pulmonary embolism.

### Case 2

**Fig. 2:**
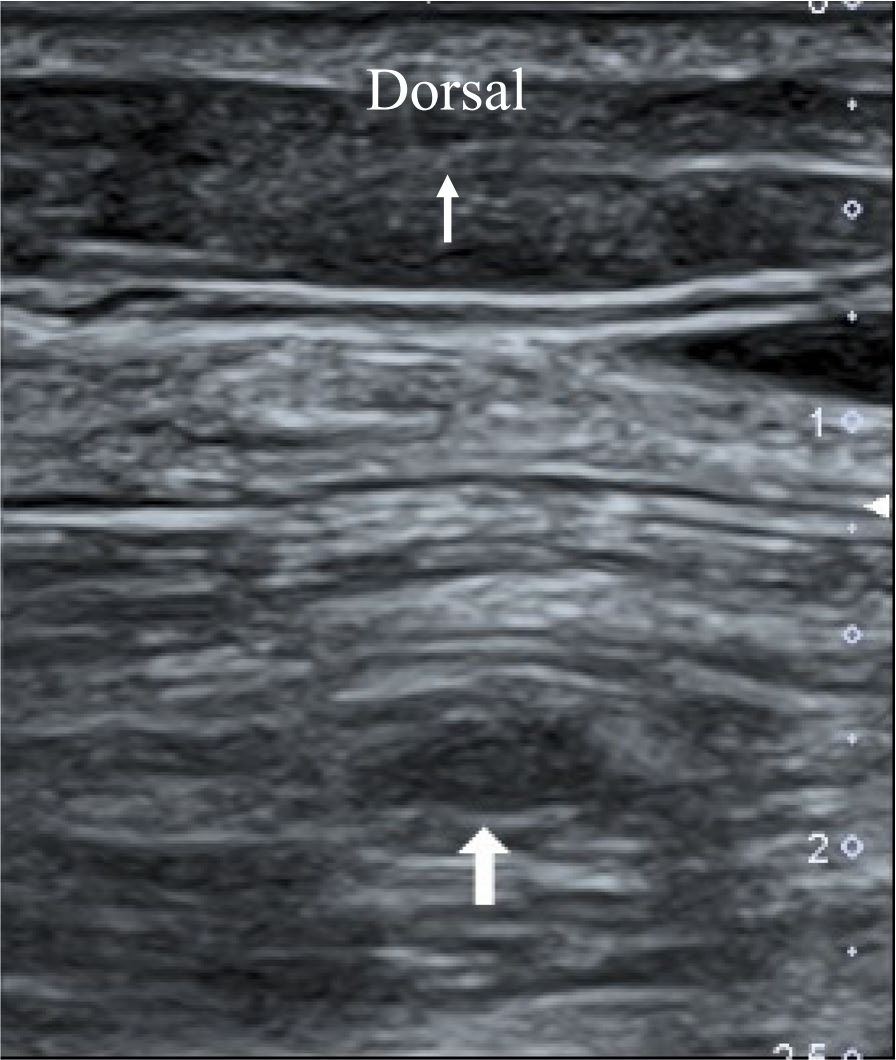
Ultrasound image of the left lower leg in case 2. An oval-shaped mass (large white arrow) is detectable in the soleal vein, which is not collapsed by compression from the surface of the calf. A small white arrow shows the direction of this image

A 94-year-old female (150 cm, 50 kg) was admitted to our hospital because of a fracture of the right neck of the femur due to falling. Her past history included a fracture of the left neck of the femur, hypertension, and dementia. Hypertension was controlled with medications. Also, she took raloxifene for osteoporosis. Osteosynthesis was emergently scheduled under spinal anesthesia. Preoperative ultrasound examination of lower extremities showed no DVT. Spinal anesthesia was induced and maintained with bupivacaine. Monitoring in the operating room included ECG, noninvasive blood pressure, percutaneous oxygen saturation, and rectal temperature. The operation was uneventful and the patient was returned to the ward. The surgery duration was 39 min. Ultrasound examination of lower extremities on the seventh postoperative day showed DVT in the left soleal vein (Fig. 2). We also started anticoagulation therapy with heparin immediately, resulting in early lysis of DVT with no serious consequences such as pulmonary embolism.

### Supplementary data

Since the two cases reported above underwent orthopedic surgery for the lower extremity, we reviewed anesthesia records for 3 years (from January 2020 to December 2022) and enrolled aged (> 65 years) female patients who underwent an emergent orthopedic operation for lower extremity while taking SERMs before the operation. Finally, we found eight female patients including the two cases above mentioned during the study period. In our hospital, both preoperative and postoperative ultrasound examination for lower extremities is routinely performed, even if an emergent operation is planned. We summarized the eight patients in Table 1. Since DVT was not detected in the other six patients, the incidence of DVT in our patient population was calculated to be 25%.

**Table 1:** Data summary in patients receiving SERMs

							Ultrasound examination for lower extremities	
SERM	Age (years)	Height (cm)	Weight (kg)	Disease	Operation	Anesthesia	Before operation	After operation
Raloxifene	83	141	36	Fracture of the left tibia	ORIF	General	DVT ( −)	DVT ( −)
Raloxifene	78	147	41.8	Periprosthetic infection (left hip)	Debridement	General	DVT ( −)	DVT ( −)
Raloxifene	79	148	40	Periprosthetic infection (left hip)	Debridement	General	DVT ( −)	DVT ( −)
Raloxifene	94	150	49.8	Fracture of the neck of the right femur	Osteosynthesis	Spinal	DVT ( −)	DVT in the left soleal vein
Raloxifene	86	158	46.9	Fracture of the neck of the right femur	Artificial head replacement	General	DVT ( −)	DVT ( −)
Bazedoxifene	86	152	55.5	Fracture of the neck of the left femur	Osteosynthesis	General	DVT ( −)	DVT ( −)
Bazedoxifene	85	146	54.3	Periprosthetic infection (right knee)	Debridement	General	DVT in the left soleal vein	DVT in the right and the left soleal vein and the right femoral vein
Bazedoxifene	74	154	72	Fracture of the neck of the right femur	Artificial head replacement	General	DVT (-)	DVT ( −)

*SERMs* selective estrogen receptor modulators, *DVT* deep venous thrombosis, *ORIF* open reduction and internal fixation

## Discussion

The current two cases suggest that we have to consider the risk of DVT after an operation in female patients while taking SERMs. Since SERMs have similar pharmacological properties as oral contraceptive pills, they need to be discontinued for a certain period before operation as WHO recommended [1]. However, now, this concept is not well recognized [4]. Moreover, in an emergent operation, it is actually impossible for the patient to discontinue SERMs for a certain period before the operation.

Osteoporosis is common and femoral neck fracture due to falling is not rare in aged patients and early osteosynthesis is required to keep the patient’s quality of life. Although periprosthetic infection is not so common, it sometimes needs an emergent debridement. Thus, it is a clinically acceptable decision to perform an emergent operation on the present two patients, even if the patients were taking SERMs. Although the two patients developed or worsened existing DVT after the operation, we started anticoagulation therapy immediately, resulting in early lysis of DVT with no serious consequences. These two cases suggest that an ultrasound examination of the lower extremities after the operation may be recommended in emergent operation patients who take SERMs.

It may be well known that estrogen and SERMs produce venous thrombosis. Activation of estrogen receptors may decrease antithrombin and protein S, resulting in favoring procoagulation and impairing anticoagulation, although the potency of each medicine may be different [5]. To our knowledge, this is the first report demonstrating that DVT developed or worsened after operation in patients taking SERMs before operation and the incidence of DVT in our small population was 25%. Recent clinical studies reported that the incidence of DVT after orthopedic surgery for the lower extremity was 6.4%, 8.2%, and 7.9%, respectively [6–8]. Thus, our incidence was larger than that of these reports, suggesting that SERMs might be a risk factor for DVT after orthopedic surgeries. However, the number of patients is too small to conclude the risk of SERMs and there are many risk factors of DVT after an operation, so multi-variate analysis may be required to conclude the risk of SERMs.

## Conclusion

We showed that DVT developed or worsened after the operation in two patients taking SERMs before the operation and the incidence was 25%. Ultrasound examination for lower extremities after operation may be recommended in these populations.

## Data Availability

The data that support the findings of this report are available from the corresponding author on reasonable reason.

## Abbreviations

DVT: Deep venous thrombosis
SERMs: Selective estrogen receptor modulators
